# Graves’ disease and systemic lupus erythematosus: a Mendelian randomization study

**DOI:** 10.3389/fimmu.2024.1273358

**Published:** 2024-01-29

**Authors:** Wei Xian, Boyuan Liu, Jinjian Li, Yuxin Yang, Shubin Hong, Haipeng Xiao, Dide Wu, Yanbing Li

**Affiliations:** ^1^ Department of Endocrinology, The First Affiliated Hospital of Sun Yat-sen University, Guangzhou, Guangdong, China; ^2^ Department of Pediatric Allergy, Immunology & Rheumatology, Guangzhou Women and Children’s Medical Center, Guangzhou, Guangdong, China; ^3^ Zhongshan School of Medicine, Sun Yat Sen University, Guangzhou, Guangdong, China

**Keywords:** Graves’ disease, systemic lupus erythematosus, GWAS, causal relationship, Mendelian randomization

## Abstract

**Introduction:**

Previous observational studies have established a correlation between Graves’ disease(GD) and systemic lupus erythematosus(SLE). However, whether a causal relationship exists between these two diseases remains unknown.We utilized Mendelian randomization to infer the causal association between GD and SLE.

**Methods:**

This study employed GWAS summary statistics of GD and SLE in individuals of Asian descent. The random effect inverse variance weighted (IVW) method was utilized to aggregate the causal effect estimates of all SNPs. Cochran’s Q values were computed to evaluate the heterogeneity among instrumental variables. Sensitivity analyses such as MR-Egger method, median weighting method, leave-one-out method, and MR-PRESSO method were used to test whether there was horizontal pleiotropy of instrumental variables.

**Results:**

Our study found genetically predicted GD may increase risk of SLE (OR=1.17, 95% CI 0.99-1.40, p=0.069). Additionally, genetically predicted SLE elevated the risk of developing GD by 15% (OR=1.15, 95% CI 1.05-1.27, p= 0.004). After correcting for possible horizontal pleiotropy by excluding outlier SNPs, the results suggested that GD increased the risk of SLE (OR=1.27, 95% CI 1.09-1.48, *p* =0.018), while SLE also increased the risk of developing GD (OR=1.13, 95% CI 1.05-1.22, *p* =0.003).

**Conclusion:**

The findings of the study indicate that there may be a correlation between GD and SLE, with each potentially increasing the risk of the other. These results have important implications for the screening and treatment of patients with co-morbidities in clinical settings, as well as for further research into the molecular mechanisms underlying the relationship between GD and SLE.

## 1 Introduction

Graves’ disease(GD) is an autoimmune disease and is the most common cause of hyperthyroidism (1). Its global incidence ranges from 20 to 50 cases per 100,000 individuals, with a prevalence of approximately 1% to 1.5% (2, 3). Graves’ disease is an autoimmune disorder that primarily affects specific organs and is susceptible to comorbidities with other autoimmune conditions, including systemic lupus erythematosus (SLE), rheumatoid arthritis, type 1 diabetes, and Addison’s disease (4). According to a comprehensive case series report, a notable proportion of patients diagnosed with GD were found to have an additional autoimmune condition, such as SLE, rheumatoid arthritis, multiple sclerosis, celiac disease, type 1 diabetes, sarcoidosis, and Sjogren’s syndrome, with a prevalence of 16.7%. Furthermore, 1.5% of patients with GD were observed to have three concurrent autoimmune diseases (4).

SLE is an autoimmune disease characterized by a diverse clinical presentation that may affect one or multiple organs, including the skin, kidneys, joints, and nervous system (5). A multicenter cohort study conducted in China revealed that individuals with GD exhibited a greater susceptibility to developing SLE compared to healthy controls (6). Previous observational studies have indicated a strong correlation between GD and SLE. However, observational studies inevitably have some shortcomings that bias the inference of causality between GD and SLE.

Mendelian randomization (MR) is an analytical approach that utilizes genetic variation as an instrumental variable to draw inferences regarding the causal relationships between variable risk factors that impact population health and target outcome factors (7). The theoretical basis for Mendelian randomization is founded on Mendel’s laws of inheritance, which dictate that allele pairs segregate and are randomly distributed to offspring during gamete formation (8). The process is similar to that of a clinical randomized controlled trial, where eligible genetic variants are used as instrumental variables for subsequent causality analysis (9). Our study aims to investigate the causal relationship between GD and SLE using bidirectional Mendelian randomization based on GWAS summary data from Asian populations.

## 2 Methods and materials

### 2.1 Data sources

In this study, we extracted SNPs for GD in Asian populations from Biobank Japan (BBJ), the largest non-European population-based biobank, which contains aggregated GWAS data for approximately 200,000 individuals of Asian origin (10). BBJ has compiled GWAS data for approximately 200,000 individuals of Asian origin and has identified 47 target diseases based on their prevalence, mortality rates, and clinical significance in the Japanese population. Patients with these diseases were enrolled and followed up at 66 hospitals in 12 Japanese medical institutions between 2003 and 2018 (10). Patients who did not belong to the Asian population or had undergone bone marrow transplantation were excluded from the study. The target diseases were diagnosed by physicians at the respective partner medical institutions, relying on their observations. The corresponding medical institutions collected both DNA and serum samples from the patients (10, 11).We accessed the summary GWAS data for GD from the Japanese ENcyclopedia of GEnetic associations by Riken (JENGER; http://jenger.riken.jp/en/) and the Medical Research Council Integrative Epidemiology Open GWAS database at the University of Bristol (MRCIEU; https://gwas.mrcieu.ac.uk/).

We obtained SNP information related to SLE in Asian populations from the GWAS published by Wang et al. in 2021, which included 4222 cases and 8431 controls from Han Chinese populations in Hong Kong, Guangzhou and Central China (12). This data was utilized to investigate the causal relationship between GD and SLE in Asian populations. The cases and controls were collected by the University of Hong Kong, Hong Kong Island West Hospital Network, and Guangzhou Women’s and Children’s Medical Center, with informed consent being obtained from all participants (12). We accessed the summary GWAS data for SLE from the GWAS catalog (https://www.ebi.ac.uk/gwas/) under data number GCST90011866.

### 2.2 Instrumental variables

Genetic variation as an instrumental variable in Mendelian randomization studies needs to satisfy three basic assumptions: i) genetic variation needs to be strongly correlated with exposure factors; ii) genetic variation must not be correlated with confounding factors; and iii) there is no independent causal pathway between genetic variation and outcome except through exposure (7, 13, 14).

SNPs that were strongly correlated with exposure factors were screened as instrumental variables from publicly available GWAS database using genomic significant level (p<5×10^-8^) as the threshold (15). To avoid weak instrumental variable bias, the F-statistic of each instrumental variable SNP was calculated in this study to assess the strength of association between SNPs and exposure factors (16). SNPs with F-statistic <10 were considered as weak instrumental variables and were excluded (16). To ensure independence between instrumental variables, we clumped the selected SNPs based on the 1000 genomes reference panel with a clumping window of 10,000 kb and an r^2^ threshold of 0.01 (8, 17).

When specific target SNPs were absent in the outcome GWAS dataset, SNPs with high LD with the target SNPs in the exposure dataset were selected as proxy SNPs from the outcome GWAS dataset in this study. To ensure a strong correlation between the proxy SNPs and the target SNPs, a threshold of r^2^ = 0.8 was set, and the proxy SNPs were subsequently used in place of the target SNPs for subsequent analysis (8).

Palindromic SNPs refer to SNPs with A/T or G/C alleles. In this study, we excluded palindromic SNPs with effect allele frequency between 0.3 and 0.7 to ensure that the reference strand where the palindromic SNP is located can be inferred (8).

### 2.3 Statistical analysis

**Figure 1:**
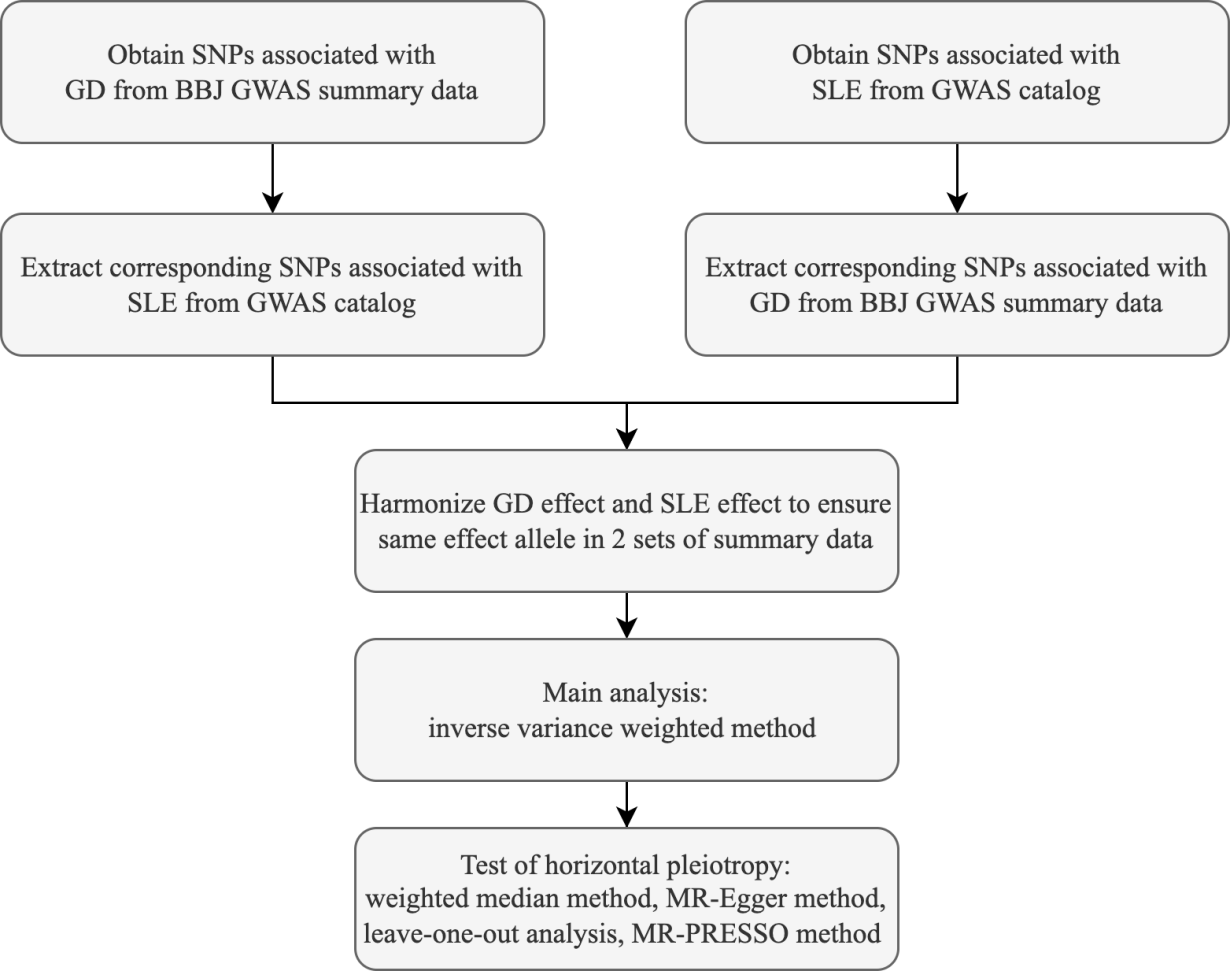
Flow chart of the 2-sample mendelian randomization study.

In this study, the causal association between GD and SLE was analyzed using the random-effects inverse variance weighted (IVW) method, which can return causal estimates corrected for heterogeneity among instrumental variables (18). Satisfaction of the second and third assumptions needs evaluation of horizontal pleiotropy (8). MR-Egger regression, median weighting, MR-PRESSO, and leave-one-out methods were operated to assess horizontal pleiotropy (8, 19, 20). MR-Egger method can be used to detect and adjust for the presence of horizontal pleiotropy, and intercept of MR-Egger regression can indicate whether horizontal pleiotropy is present (21). Cochran’s Q value was calculated to assess the heterogeneity of the causal effects among genetic variants (22). The weighted median method is able to provide a consistent estimate of the causal effect when only 50% of instrumental variables are valid (23). Mendelian Randomization Pleiotropy RESidual Sum and Outlier (MR-PRESSO) is a method for testing and identifying outliers and correcting horizontal pleiotropy (20). The leave-one-out analysis is used to test whether a particular SNP or a group of SNPs has a significant influence on the causal effect estimate and to assess the robustness of the results (8). Estimates of causal effects were reflected using the odds ratio (OR) and 95% confidence interval (CI). The main statistical analyses were performed using R software (version 4.2.1) and the R language packages TwoSampleMR (version 0.5.6), ieugwasr (version 0.1.5). Flow chart of this study is shown in **Figure 1**.

## 3 Results

Thirteen SNPs significantly related to GD were extracted from the results of BBJ. Thirty-nine SNPs significantly related to SLE were obtained from summary data of Wang et al. After clumping and removing palindromic SNPs with effect allele frequency between 0.3 and 0.7, 12 SNPs were eligible to analyze causal effect of GD on SLE, and 36 SNPs were eligible to analyze causal effect of SLE on GD. Details of SNPs associated with GD and SLE were shown in **Supplementary Tables 1–4** (24).

**Figure 2:**
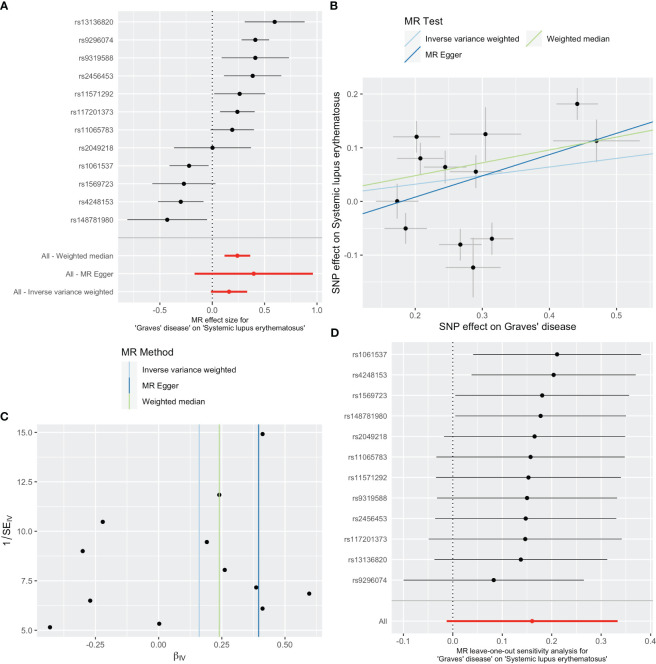
Plots of causal estimates of genetically predicted Graves’ disease on systemic lupus erythematosus. **(A)** The forest plot. **(B)** The scatter plot. **(C)** The funnel plot. **(D)** The leave-one-out plot.

As shown in **Table 1**, GD slightly increased the risk of SLE as an exposure factor (OR=1.17, 95% CI 0.99-1.40, *p* =0.069). Moreover, the median weighting analysis showed a more significant causal relationship (OR=1.27, 95% CI 1.12-1.44, *p <*0.001). The MR-Egger intercept did not indicate significant horizontal pleiotropy bias (*p* =0.410). Cochran’s Q value revealed heterogeneity among the causal effects derived from different GD instrumental variables(*p <*0.001). The forest plots, scatter plots, and funnel plots suggested that there was heterogeneity between the effect estimates of instrumental variables for GD on SLE. In sensitivity analysis, the leave-one-out method indicated that removing GD-related SNPs one by one did not significantly alter the causal effect (**Figure 2**).

**Table 1:** Mendelian randomization estimates of genetically predicted Graves’ disease on systemic lupus erythematosus.

Outcome	Method	OR	95% CI	p	Cochran’s Q (p)	Intercept (p)
SLE	IVW	1.17	0.99-1.40	0.069	80.50(<0.001)	
	MR-Egger	1.49	0.84-2.61	0.200		-0.07(0.410)
	WM	1.27	1.12-1.44	<0.001		

**Figure 3:**
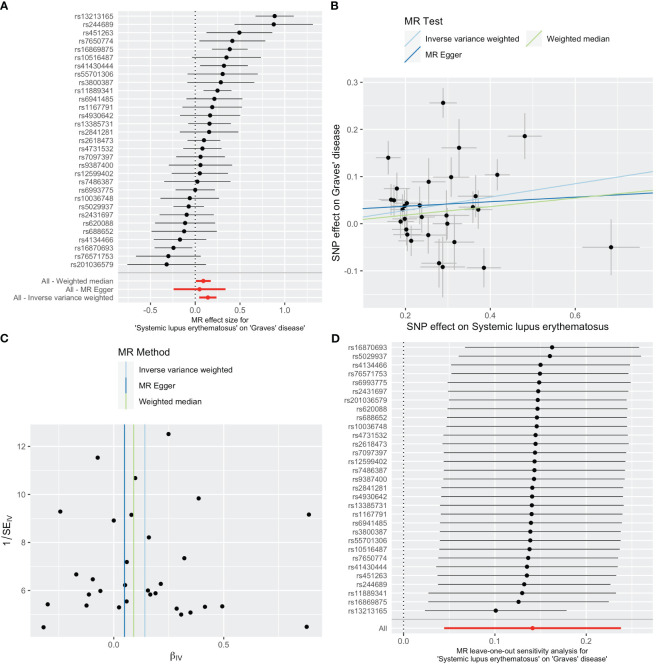
Plots of causal estimates of genetically predicted systemic lupus erythematosus on Graves’ disease. **(A)** The forest plot. **(B)** The scatter plot. **(C)** The funnel plot. **(D)** The leave-one-out plot.

As shown in **Table 2**, SLE increased the risk of GD when GD was the outcome. SLE raised the risk of developing GD by 15% (OR=1.15, 95% CI 1.05-1.27, *p* =0.004). The weighted median analysis showed a consistent causal effect (OR=1.09, 95% CI 1.01-1.19, *p* =0.038). The MR-Egger intercept was not significant (*p* =0.510), and there was heterogeneity among the causal effect estimates derived from different SLE instrumental variables (*p <*0.001).The forest plot and scatter plot indicated that SNPs rs13213165 and rs244689 had more prominent effects. In sensitivity analysis, the leave-one-out method indicated that removing the SNPs associated with SLE with prominent effects one by one did not significantly change the overall causal effect (**Figure 3**).

**Table 2:** Mendelian randomization estimates of genetically predicted systemic lupus erythematosus on Graves’ disease.

Exposure	Method	OR	95% CI	p	Cochran’s Q (p)	Intercept (p)
SLE	IVW	1.15	1.05-1.27	0.004	119.71(<0.001)	
	MR-Egger	1.05	0.79-1.40	0.745		0.03(0.510)
	WM	1.09	1.01-1.19	0.038		

The MR-PRESSO overall test and outlier test suggested that there might be outlier SNPs influencing the causal effect estimates between GD and SLE (**Table 3**). After correcting for possible horizontal pleiotropy by excluding outlier SNPs, the effect of GD on SLE (Distortion *p* =0.303) and the causal effect of SLE on GD (Distortion *p* =0.517) were not significantly different from those before correction (**Table 3**). The corrected results suggested that GD increased the risk of SLE (OR=1.27, 95% CI 1.09-1.48, *p* =0.018), while SLE also increased the risk of developing GD (OR=1.13, 95% CI 1.05-1.22, *p* =0.003).

**Table 3:** MR-PRESSO results of the causal relationship between genetically predicted Graves’ disease and systemic lupus erythematosus.

Exposure	Outcome	Global p	Outliers	Original OR (95%CI)	Original p	Corrected OR (95%CI)	Corrected p
GD	SLE	<0.001	4	1.17(0.99-1.39)	0.096	1.27(1.09-1.48)	0.018
SLE	GD	<0.001	2	1.15(1.05-1.27)	0.007	1.13(1.05-1.22)	0.003

## 4 Discussion

In this study, we identified a bidirectional causal association between genetically predicted GD and SLE. Prior epidemiological investigations have indicated the co-occurrence of GD and SLE in certain patients, and a multicenter cohort study has reported a higher risk of SLE in individuals with GD compared to those without GD (6). Patients with SLE may exhibit clinical indications of thyroid dysfunction and comorbidity with GD (4, 25). The causal association between GD and SLE found in our study further confirms these previous epidemiological findings.

The development of SLE and GD may be influenced by mutations in HLA genes. A meta-analysis conducted recently revealed that mutations in HLA-DR3 and HLA-DR15 significantly increased the likelihood of developing SLE, thereby indicating the potential role of the HLA-DRB1 gene as a susceptibility gene for SLE (26). A study by Zawadzka-Starczewska et al. also found that HLA-DRB1 gene mutations were associated with the risk of developing GD (27). The presence of mutated HLA genes can result in alterations to the HLA complex on antigen-presenting cells, thereby influencing the interaction between B and T cells. This situation can lead to the dysregulation of self-reactive B cells and the production of autoantibodies, which may serve as mediators of the causal effect of SLE on GD.

In addition to HLA genes, non-HLA genes may also play an important role in the interaction between SLE and GD. Cytotoxic T-lymphocyte-associated protein 4 (CTLA-4) is a protein receptor that functions as an immune checkpoint to downregulate immune responses by binding to ligands CD80 and CD86 on the surface of antigen-presenting cells, thereby regulating T-cell activation and proliferation (28). Mutations in the CTLA-4 gene have been associated with both SLE and GD. A meta-analysis indicated that CTLA-4 gene polymorphisms in Asian populations were associated with the risk of developing SLE (29). A study by Lee et al. found that multiple single nucleotide polymorphisms of CTLA-4 were associated with GD and autoimmune thyroid diseases such as Hashimoto’s thyroiditis (30). Mutations in the CTLA-4 gene may lead to a deficiency in regulatory T-cell downregulation of immune responses, resulting in T-cell hyperactivation and thus triggering autoimmune diseases. They may also play a mediating role in the relationship between GD and SLE.

The PTPN22 gene is another common genetic susceptibility locus for GD and SLE. A meta-analysis conducted by Hu et al. suggested that the SNP rs2476601 mutation in the PTPN gene was linked to an elevated risk of developing SLE in European and American populations (31). Further studies suggested that downregulation of PTPN22 mRNA expression levels was associated with higher SLE activity and more severe lupus nephritis (32). A study conducted by Ichimura et al. found a higher susceptibility to GD in a Japanese population with mutations in the PTPN22 gene (33). The presence of polymorphisms in the PTPN22 gene may affect the functions of T, B, and myeloid cells, as well as regulate their cytokine secretion, potentially influencing the onset of SLE. These genetic variations may play a role in the development of autoimmune disorders, including SLE and GD, and may be implicated in the causal relationship between them (34).

Both SLE and GD are characterized by immune regulation abnormalities, which may be closely associated with type I interferon (IFN-I) (35). Type I interferons, including IFN-α, IFN-β, IFN-ϵ, IFN-κ, etc., are cytokines that play crucial roles in inflammation, immune regulation, tumor cell recognition, and T-cell responses. In the context of autoimmune diseases, IFN-I can contribute to the development and progression of SLE by promoting antigen presentation and lymphocyte responses, as well as inducing chemokine expression. They can also promote cell activation and enhance responsiveness to inflammatory factors (36). IFN-I enhances B cell activation, differentiation, proliferation and antibody production, and may induce the expression of thyroid-stimulating hormone receptors. Thus, it may have an impact on the development of GD (37). Several type I interferon-related genes, including STAT4, IRF5, IFIH1, and PLZF, have been found in previous studies to be associated with both GD and SLE (38).

Furthermore, aside from aberrant immune regulation, SLE and GD may share a common pathogenesis with regards to autoantibodies that result in tissue damage. A meta-analysis conducted by Pan et al. indicated that levels of thyroid peroxidase antibodies (TPOAb) and thyroglobulin antibodies (TgAb) were considerably higher in SLE patients compared to the non-affected population, implying a correlation between SLE and autoimmune thyroid disease (39). This is consistent with the causal association results of our study. A study conducted by Lanzolla et al. indicated that the presence of antinuclear antibodies was observed in around 80% of patients diagnosed with GD. This finding suggested that GD may potentially influence the development of SLE through the autoantibody pathway (40). Furthermore, a shared immunological pathway in the development of SLE and GD is suggested by the overlapping presence of specific chemokines and cytokines. Recent researches have highlighted that the interaction between CXCL10 and CXCR3 in the T helper 1 immune response is pivotal in the etiology of both GD and SLE (41, 42). Moreover, it was reported that elevated levels of IL-37 was positively correlated with the concentration of TRAb and the severity of SLE, proposing that IL-37 could play a significant role in the co-occurrence of GD and SLE (43).

In addition, environmental factors may play a role in triggering or exacerbating immune disorders in GD and SLE. A previous study conducted by Parks et al. found that air pollution and dust exposure may elevate the risk of SLE through epigenetic alterations, increased oxidative stress, and increased secretion of systemic inflammatory factors (44). Similarly, a study by Kim et al. in a Korean cohort proposed a potential association between air pollution and aberrant thyroid function in the general populace (45). Changes in the composition of the microbiota are linked to impaired intestinal barrier function and dysregulation of the mucosal immune system, which may be connected to the development of GD and SLE (46, 47). Smoking may also have an impact on the development of SLE and GD through epigenetic and systemic inflammatory responses (48, 49). Infection with some viruses such as EBV and CMV is also associated with the risk of developing SLE and GD (49, 50). The postulated potential mechanism is that EBV persists in B cells and is activated from time to time, possibly by stimulating TRAb-producing B cells to promote TRAb production in patients with GD (49). On the other hand, both lysed and latent EBV proteins elicit strong T- and B-cell responses and the EBV virus itself induces a series of changes in the body’s immune system, which may induce systemic lupus erythematosus (51).

Our study has the following strengths. First, it builds on a large body of previous epidemiological evidence suggesting a co-morbid relationship between GD and SLE. It is the first to explore the causal relationship between GD and SLE at the genetic level using the Mendelian randomization method and to determine the direction of the causal effect. Second, it uses the results of large-scale GWAS studies for analysis and provides a high level of evidence. Third, it extracts GWAS summary data from an Asian population, which has a genetic background similar to ours.

The limitations of this study include following aspects. First, it only analyzes data from the genetic background of Asian populations, and its applicability to European, American and African populations still needs further validation. Second, it does not further analyze the molecular mechanisms of GD and SLE, so more studies are still needed to explore the molecular mechanisms in the causal effects of GD and SLE. This would help to better select targets for screening and treatment.

## 5 Conclusion

Genetically predicted GD increases the risk of developing SLE, and vice versa. The results of our study provide a new basis for screening and treatment of co-morbidities in clinical practice. Patients with GD who have a longer duration or new non-specific symptoms should be screened for SLE to facilitate timely diagnosis and treatment and avoid delay or exacerbation of the disease. At the same time, it is also necessary to monitor thyroid function during the diagnosis and treatment of SLE, so as to adjust the treatment plan accordingly and ensure the quality of life of patients.

## Data availability statement

The original contributions presented in the study are included in the article/**Supplementary Material**. Further inquiries can be directed to the corresponding authors.

## Ethics statement

Ethical approval was not required for the study involving humans in accordance with the local legislation and institutional requirements. Written informed consent to participate in this study was not required from the participants or the participants’ legal guardians/next of kin in accordance with the national legislation and the institutional requirements.

## Author contributions

WX: Conceptualization, Formal analysis, Methodology, Software, Validation, Visualization, Writing – original draft, Writing – review & editing, Data curation. BL: Conceptualization, Writing – original draft, Writing – review & editing, Data curation, Formal analysis, Methodology, Software, Validation, Visualization. JL: Conceptualization, Formal analysis, Methodology, Validation, Writing – original draft, Writing – review & editing. YY: Validation, Visualization, Writing – original draft. SH: Formal analysis, Methodology, Validation, Visualization, Writing – original draft. HX: Data curation, Supervision, Validation, Visualization, Writing – original draft. DW: Conceptualization, Formal analysis, Funding acquisition, Methodology, Supervision, Validation, Writing – original draft, Writing – review & editing. YL: Conceptualization, Investigation, Project administration, Supervision, Writing – original draft, Writing – review & editing.
