# Supplementary material for: Graves’ disease and systemic lupus erythematosus: a Mendelian randomization study
Source: Front Immunol. 2024 Jan 29;15:1273358. doi: 10.3389/fimmu.2024.1273358 (PMC10863043; doi:10.3389/fimmu.2024.1273358)
Supplement: Supplementary file 1 [file Table_1.docx]

Supplementary table 1. Details of SNPs associated with Grave’s disease

| SNP | Chr | Position | Gene | EA | NEA | EAF | β | SE | *P* | F statistic |
| --- | --- | --- | --- | --- | --- | --- | --- | --- | --- | --- |
| rs117201373 | 1 | 160419940 | . | G | A | 0.0653 | 0.4701 | 0.0646 | 3.29E-13 | 53.03 |
| rs11571292 | 2 | 204720139 | . | A | G | 0.6136 | 0.2447 | 0.0317 | 1.10E-14 | 59.70 |
| rs2049218 | 3 | 188122978 | LPP | T | C | 0.3886 | -0.1731 | 0.0317 | 4.89E-08 | 29.76 |
| rs13136820 | 4 | 40307564 | LINC02265 | T | C | 0.7193 | -0.2021 | 0.0351 | 8.40E-09 | 33.18 |
| rs1061537 | 6 | 29937795 | . | A | G | 0.6236 | -0.3143 | 0.0321 | 1.10E-22 | 96.09 |
| rs148781980 | 6 | 32667577 | . | G | A | 0.1773 | 0.2863 | 0.0408 | 2.32E-12 | 49.19 |
| rs9296074 | 6 | 33042598 | HLA-DPB1, HLA-DPA1 | G | A | 0.4689 | 0.4415 | 0.0310 | 4.91E-46 | 202.88 |
| rs4248153 | 6 | 31002527 | MUC22 | G | A | 0.6205 | -0.2673 | 0.0318 | 3.91E-17 | 70.82 |
| rs2456453 | 8 | 128201359 | CASC19 | T | C | 0.3406 | -0.2077 | 0.0348 | 2.35E-09 | 35.66 |
| rs11065783 | 12 | 111396249 | LOC105369980 | G | A | 0.2695 | 0.2904 | 0.0388 | 7.23E-14 | 56.01 |
| rs4903961 | 14 | 81462649 | TSHR | G | C | 0.6134 | 0.2344 | 0.0319 | 1.96E-13 | 54.05 |
| rs9319588 | 16 | 30930983 | FBXL19-AS1 | T | C | 0.9055 | -0.3047 | 0.0531 | 9.63E-09 | 32.91 |
| rs1569723 | 20 | 44742064 | . | A | C | 0.6152 | 0.1857 | 0.0316 | 4.06E-09 | 34.59 |

Abbreviations: Chr, Chromosome; SNP, single nucleotide polymorphism; EA, effect allele; NEA, non-effect allele; EAF, effect allele frequency; β, effect size; SE, standard error.

Supplementary table 2. Details of SNPs associated with Graves’ disease and association with systemic lupus erythematosus.

| SNP | Chr | Position | EA | NEA | β | SE | *P* |
| --- | --- | --- | --- | --- | --- | --- | --- |
| rs117201373 | 1 | 160419940 | G | A | 0.1128 | 0.0397 | 4.53E-03 |
| rs11571292 | 2 | 204720139 | A | G | 0.0640 | 0.0304 | 3.55E-02 |
| rs2049218 | 3 | 188122978 | T | C | -0.0004 | 0.0325 | 9.91E-01 |
| rs13136820 | 4 | 40307564 | T | C | -0.1204 | 0.0295 | 4.47E-05 |
| rs4248153 | 6 | 31002527 | G | A | 0.0803 | 0.0297 | 6.93E-03 |
| rs148781980 | 6 | 32667577 | G | A | -0.1231 | 0.0556 | 2.69E-02 |
| rs9296074 | 6 | 33042598 | G | A | 0.1816 | 0.0296 | 8.49E-10 |
| rs1061537 | 6 | 29937795 | A | G | 0.0695 | 0.0300 | 2.06E-02 |
| rs2456453 | 8 | 128201359 | T | C | -0.0802 | 0.0290 | 5.60E-03 |
| rs11065783 | 12 | 111396249 | G | A | 0.0554 | 0.0307 | 7.14E-02 |
| rs4903961 | 14 | 81462649 | G | C | 0.0056 | 0.0297 | 8.50E-01 |
| rs9319588 | 16 | 30930983 | T | C | -0.1253 | 0.0500 | 1.22E-02 |
| rs1569723 | 20 | 44742064 | A | C | -0.0503 | 0.0286 | 7.91E-02 |

Effect allele frequency is not available in GWAS summary data of systemic lupus erythematosus.

Abbreviation: Chr, Chromosome; SNP, single nucleotide polymorphism; EA, effect allele; NEA, non-effect allele; EAF, effect allele frequency; β, effect size; SE, standard error.

Supplementary table 3. Details of SNPs associated with systemic lupus erythematosus.

| SNP | Chr | Position | Gene | EA | NEA | β | SE | *P* | F statistic |
| --- | --- | --- | --- | --- | --- | --- | --- | --- | --- |
| rs4844538 | 1 | 206642720 | IKBKE | T | A | -0.1883 | 0.0322 | 4.81E-09 | 34.20 |
| rs201036579 | 1 | 157499390 | FCRL5 | C | T | -0.2878 | 0.0527 | 4.85E-08 | 29.82 |
| rs41430444 | 2 | 191878487 | STAT1 | C | T | -0.3077 | 0.0428 | 6.57E-13 | 51.69 |
| rs11889341 | 2 | 191943742 | STAT4 | T | C | 0.4164 | 0.0295 | 3.33E-45 | 199.24 |
| rs13385731 | 2 | 33701890 | RASGRP3 | C | T | -0.3654 | 0.0421 | 4.19E-18 | 75.33 |
| rs7650774 | 3 | 119205050 | POGLUT1 | C | T | -0.1798 | 0.0306 | 4.07E-09 | 34.53 |
| rs10516487 | 4 | 102751076 | BANK1 | A | G | -0.2547 | 0.0398 | 1.64E-10 | 40.95 |
| rs244689 | 5 | 133422816 | LOC105379185 | G | A | -0.1595 | 0.0291 | 4.29E-08 | 30.04 |
| rs10036748 | 5 | 150458146 | TNIP1 | T | C | 0.2021 | 0.0338 | 2.29E-09 | 35.75 |
| rs2431697 | 5 | 159879978 | . | C | T | -0.2538 | 0.0451 | 1.84E-08 | 31.67 |
| rs16870693 | 6 | 32711691 | HLA-DQA2 | A | C | 0.3849 | 0.0403 | 1.37E-21 | 91.22 |
| rs451263 | 6 | 34549498 | . | A | G | 0.3263 | 0.0414 | 3.02E-15 | 62.12 |
| rs6941485 | 6 | 250619 | . | G | A | 0.2034 | 0.0314 | 9.53E-11 | 41.96 |
| rs9269627 | 6 | 32545604 | . | T | C | -0.6737 | 0.0347 | 9.32E-84 | 376.94 |
| rs13213165 | 6 | 33039729 | HLA-DPA1 | G | T | 0.2888 | 0.0322 | 3.13E-19 | 80.44 |
| rs3800387 | 6 | 35186501 | SCUBE3 | A | G | 0.1731 | 0.0303 | 1.08E-08 | 32.64 |
| rs9387400 | 6 | 116694120 | DSE,LOC100287467 | A | C | -0.2967 | 0.0536 | 3.14E-08 | 30.64 |
| rs16869875 | 6 | 32192217 | NOTCH4 | T | C | 0.4811 | 0.0400 | 3.01E-33 | 144.66 |
| rs5029937 | 6 | 138195151 | TNFAIP3 | T | G | 0.6844 | 0.0676 | 4.32E-24 | 102.50 |
| rs4134466 | 6 | 106577368 | . | G | A | -0.2136 | 0.0306 | 3.09E-12 | 48.73 |
| rs688652 | 6 | 138141724 | . | C | T | -0.3154 | 0.0468 | 1.61E-11 | 45.42 |
| rs11185603 | 7 | 50306810 | . | G | C | -0.2703 | 0.0327 | 1.34E-16 | 68.33 |
| rs76571753 | 7 | 73974915 | GTF2IRD1 | T | G | 0.2792 | 0.0414 | 1.46E-11 | 45.48 |
| rs377080180 | 7 | 74121005 | GTF2I,LOC101926943 | AT | A | 0.5052 | 0.0842 | 1.99E-09 | 36.00 |
| rs4731532 | 7 | 128572766 | . | A | G | 0.3716 | 0.0334 | 8.15E-29 | 123.78 |
| rs6993775 | 8 | 11369989 | BLK | T | G | 0.2987 | 0.0346 | 6.06E-18 | 74.53 |
| rs2618473 | 8 | 11344127 | . | T | C | 0.3590 | 0.0339 | 2.89E-26 | 112.15 |
| rs7097397 | 10 | 50025396 | WDFY4 | A | G | -0.2385 | 0.0310 | 1.32E-14 | 59.19 |
| rs4930642 | 11 | 68816370 | TPCN2 | G | A | -0.2336 | 0.0344 | 1.10E-11 | 46.11 |
| rs12575600 | 11 | 128324869 | LOC105369566 | G | C | 0.2760 | 0.0293 | 4.12E-21 | 88.73 |
| rs620088 | 11 | 65508986 | . | A | G | -0.2044 | 0.0334 | 9.38E-10 | 37.45 |
| rs7486387 | 12 | 12866911 | GPR19 | A | G | -0.1886 | 0.0314 | 1.98E-09 | 36.08 |
| rs11059928 | 12 | 129296103 | SLC15A4 | T | A | 0.2888 | 0.0348 | 1.15E-16 | 68.87 |
| rs2841281 | 14 | 105394669 | PLD4 | T | C | 0.1933 | 0.0292 | 3.56E-11 | 43.82 |
| rs142105922 | 16 | 86002593 | . | AAAT | A | -0.4029 | 0.0592 | 9.73E-12 | 46.32 |
| rs12599402 | 16 | 11189888 | CLEC16A | C | T | -0.1983 | 0.0292 | 1.02E-11 | 46.12 |
| rs55701306 | 17 | 16842447 | TNFRSF13B | T | C | 0.1662 | 0.0293 | 1.38E-08 | 32.18 |
| rs5749502 | 22 | 21945096 | UBE2L3 | A | T | 0.2173 | 0.0289 | 5.43E-14 | 56.54 |

Effect allele frequency is not available in GWAS summary data of systemic lupus erythematosus.

Continue of supplementary table 3

Abbreviation: Chr, Chromosome; SNP, single nucleotide polymorphism; EA, effect allele; NEA, non-effect allele; β, effect size; SE, standard error.

Supplementary table 4. Details of SNPs associated with systemic lupus erythematosus and association with Graves’ disease.

| SNP | Chr | Position | EA | NEA | β | SE | EAF | *P* |
| --- | --- | --- | --- | --- | --- | --- | --- | --- |
| rs201036579 | 1 | 157499390 | C | T | 0.0919 | 0.0645 | 0.0625 | 1.54E-01 |
| rs4844538 | 1 | 206642720 | T | A | -0.0120 | 0.0366 | 0.2701 | 7.44E-01 |
| rs41430444 | 2 | 191878487 | C | T | -0.0987 | 0.0419 | 0.1636 | 1.85E-02 |
| rs11889341 | 2 | 191943742 | T | C | 0.1036 | 0.0333 | 0.3061 | 1.84E-03 |
| rs13385731 | 2 | 33701890 | C | T | -0.0583 | 0.0445 | 0.1404 | 1.90E-01 |
| rs7650774 | 3 | 119205050 | C | T | -0.0745 | 0.0338 | 0.2941 | 2.76E-02 |
| rs10516487 | 4 | 102751076 | A | G | -0.0888 | 0.0502 | 0.1054 | 7.66E-02 |
| rs10036748 | 5 | 150458146 | T | C | -0.0123 | 0.0338 | 0.7080 | 7.16E-01 |
| rs244689 | 5 | 133422816 | G | A | -0.1399 | 0.0356 | 0.6769 | 8.46E-05 |
| rs2431697 | 5 | 159879978 | C | T | 0.0242 | 0.0393 | 0.1882 | 5.38E-01 |
| rs5029937 | 6 | 138195151 | T | G | -0.0500 | 0.0594 | 0.0717 | 3.99E-01 |
| rs9387400 | 6 | 116694120 | A | C | -0.0173 | 0.0536 | 0.9079 | 7.46E-01 |
| rs6941485 | 6 | 250619 | G | A | 0.0435 | 0.0324 | 0.4057 | 1.80E-01 |
| rs13213165 | 6 | 33039729 | G | T | 0.2562 | 0.0315 | 0.6059 | 4.48E-16 |
| rs16869875 | 6 | 32192217 | T | C | 0.1856 | 0.0489 | 0.1109 | 1.48E-04 |
| rs16870693 | 6 | 32711691 | A | C | -0.0936 | 0.0415 | 0.1704 | 2.40E-02 |
| rs451263 | 6 | 34549498 | A | G | 0.1609 | 0.0612 | 0.0698 | 8.52E-03 |
| rs3800387 | 6 | 35186501 | A | G | 0.0495 | 0.0330 | 0.3163 | 1.34E-01 |
| rs4134466 | 6 | 106577368 | G | A | 0.0364 | 0.0320 | 0.6338 | 2.56E-01 |
| rs688652 | 6 | 138141724 | C | T | 0.0391 | 0.0588 | 0.9262 | 5.06E-01 |
| rs11185603 | 7 | 50306810 | G | C | 0.0933 | 0.0308 | 0.4766 | 2.41E-03 |
| rs76571753 | 7 | 73974915 | T | G | -0.0839 | 0.0515 | 0.1313 | 1.04E-01 |
| rs1167791 | 7 | 75170570 | T | C | 0.0376 | 0.0337 | 0.6806 | 2.64E-01 |
| rs4731532 | 7 | 128572766 | A | G | 0.0298 | 0.0406 | 0.1882 | 4.63E-01 |
| rs2618473 | 8 | 11344127 | T | C | 0.0350 | 0.0336 | 0.6998 | 2.97E-01 |
| rs6993775 | 8 | 11369989 | T | G | 0.0000 | 0.0335 | 0.7023 | 1.00E+00 |
| rs7097397 | 10 | 50025396 | A | G | -0.0142 | 0.0332 | 0.6786 | 6.70E-01 |
| rs12575600 | 11 | 128324869 | G | C | 0.0358 | 0.0314 | 0.3917 | 2.55E-01 |
| rs620088 | 11 | 65508986 | A | G | 0.0231 | 0.0351 | 0.2629 | 5.10E-01 |
| rs4930642 | 11 | 68816370 | G | A | -0.0388 | 0.0401 | 0.7796 | 3.33E-01 |
| rs7486387 | 12 | 12866911 | A | G | -0.0045 | 0.0356 | 0.2688 | 8.99E-01 |
| rs11059928 | 12 | 129296103 | T | A | -0.0016 | 0.0417 | 0.1642 | 9.69E-01 |
| rs2841281 | 14 | 105394669 | T | C | 0.0300 | 0.0322 | 0.6258 | 3.52E-01 |
| rs12599402 | 16 | 11189888 | C | T | -0.0102 | 0.0319 | 0.3724 | 7.49E-01 |
| rs55701306 | 17 | 16842447 | T | C | 0.0509 | 0.0333 | 0.3479 | 1.26E-01 |
| rs5749502 | 22 | 21945096 | A | T | 0.0650 | 0.0332 | 0.3608 | 5.01E-02 |

Abbreviation: Chr, Chromosome; SNP, single nucleotide polymorphism; EA, effect allele; NEA, non-effect allele; EAF, effect allele frequency; β, effect size; SE, standard error.

Continue of supplementary table 4

16
